# Exploring caspase functions in mouse models

**DOI:** 10.1007/s10495-024-01976-z

**Published:** 2024-06-02

**Authors:** Eva Svandova, Barbora Vesela, Eva Janeckova, Yang Chai, Eva Matalova

**Affiliations:** 1 Laboratory of Odontogenesis and Osteogenesis, Institute of Animal Physiology and Genetic, Brno, Czech Republic; 2 Center for Craniofacial Molecular Biology, University of Southern California, Los Angeles, USA; 3 Department of Physiology, University of Veterinary Sciences, Brno, Czech Republic

**Keywords:** Caspases, Apoptotic, Non-apoptoic, Deficiency, Animal model, Mouse

## Abstract

Caspases are enzymes with protease activity. Despite being known for more than three decades, caspase investigation still yields surprising and fascinating information. Initially associated with cell death and inflammation, their functions have gradually been revealed to extend beyond, targeting pathways such as cell proliferation, migration, and differentiation. These processes are also associated with disease mechanisms, positioning caspases as potential targets for numerous pathologies including inflammatory, neurological, metabolic, or oncological conditions. While in vitro studies play a crucial role in elucidating molecular pathways, they lack the context of the body’s complexity. Therefore, laboratory animals are an indispensable part of successfully understanding and applying caspase networks. This paper aims to summarize and discuss recent knowledge, understanding, and challenges in caspase knock-out mice.

## Introduction

Caspases, also known as **c**ysteine-dependent **asp**artate-specific prote**ases** (alternatively cysteine-aspartic proteases or cysteine aspartic acid proteases), are enzymes that utilize the sulfur atom in cysteine to catalyze cleavage reaction. Together with the serine protease granzyme B, caspases display specificity for Asp in the P1 position of the caspase recognition motif when processing their substrates [1]. The caspase family is highly evolutionary conserved, underscoring its importance across various organisms [2]. Research on caspases began with the identification of protease activity that generates mature interleukin (IL)-1β from its precursor in extracts of human monocytes, where it plays a crucial role in regulating inflammatory responses [3]. Few years later, unusual cleavage at Asp-X bonds of the interleukin-1*β*-converting enzyme (ICE), also known as caspase-1, was identified [4] and specified in 1992 [5, 6]. In 1993, the *C. elegans* Cell death protein-3 (CED-3) and mammalian ICE similarity was revealed and associated with programmed cell death - apoptosis. Along with caspase-1, caspase-2 was one of the first discovered mammalian homologues of CED-3 [7]. By 1998, crucial protein components that participate in apoptosis were defined in humans and laboratory mice [8, 9]. Further research brought discovery of members of caspase family in vertebrates standing behind apoptosis or inflammation, function of which remains mostly unexplained, this applies for caspase-15-18 [10, 11].

**Fig. 1:**
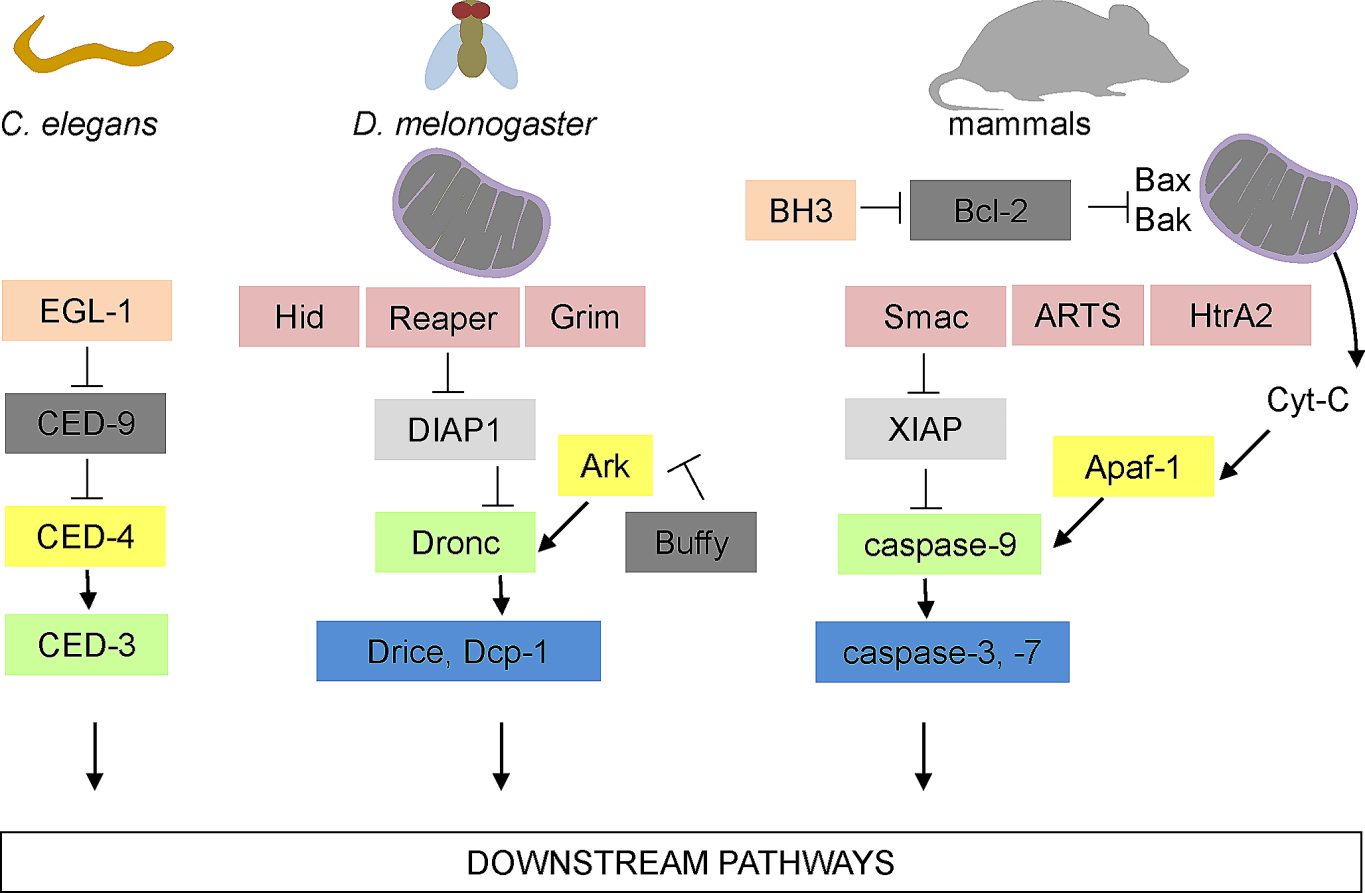
Conserved caspase signalling cascades in eukaryotic organisms. In *C. elegans*, the antagonist EGL-1 inhibits CED-9, leading to the release of CED-4 from the CED-9–CED-4 complex. This liberation promotes the activation of CED-3. In *Drosophila*, the inhibitors of apoptosis (IAPs) Reaper, Hid, and Grim facilitate the degradation of DIAP1, thereby freeing Drice and Dcp-1. This process also involves the interaction of Dronc with Ark and the formation of the apoptosome, which activates executioner caspases. The activation of the apoptosome might be regulated by proteins such as Buffy. In mammals, Bcl-2 and BH3-only proteins regulate BAX- and BAK-dependent release of cytochrome c from the mitochondria. Cytochrome-c then binds to APAF1 to form the apoptosome. In parallel, IAP antagonists, including DIABLO, HTRA2 and ARTS, translocate from the mitochondria and release caspases from their negative regulation by IAPs. Caspase-9 is subsequently liberated from XIAP and activated by the apoptosome, triggering executioner caspases-3 and − 7. Green: caspase-9 like, yellow: Apaf-1 like, blue: executor caspases, dark grey: Bcl-2 like, light grey: apoptotic inhibitors, pink: BH3-only like, purple: IAP binding. Figure based on Bell and Megeney [284], Fuchs and Steller [285]

The presence of specific caspases varies among species (Table 1). This variability is evident when comparing the mouse model to the humans. For instance, mouse caspase-11 is considered an orthologue of human caspase-4 and caspase-5, sharing 68% and 47% of amino acid sequences, respectively [12]. Mice express full length of caspase-12 [13], while primarily a truncated form is present in humans [14]. Conversely, mice lack caspase-5 [15] and -10 [16] compared to humans. Notably, despite these differences, the cascade of molecular caspase pathways is conserved across eukaryotes (Fig. 1).

**Table 1:** Comparison of caspases in different species with focus on classical caspase categorisation. In category „others“ the caspase either does not fit groups above or have not yet been specified. * differentiation of keratinocytes [259], **regulates non-canonical pathway of apoptosis [291], *** blocks CED-3 and apoptosis in germ cells [292], **** blocks CED-3 and apoptosis in somatic cells [292]

	human	mouse M. musculus	zebrafish D. rerio	fruitfly D. melanogaster	worm C. elegans
number of caspases	13	11	19	7	4
inflammatory caspases	-1, -4, -5 -12	-1, -11, -12	-1, -19a, -19b, -23		
initiator caspases	-2, -8, -9, -10	-2, -8, -9	-2, -8a, -8b, -9, -10, -20, -22	Dredd, Dronc, Strica	
executor caspases	-3, -6, -7	-3, -6, -7	-3a, -3b, -6a, -6b, -6c,-7, -21	Drice, Dcp-1, Decay, Dam	CED-3
others	-14*, -16	-14*, − 16	-17		CSP-1**, CSP-2***, and CSP-3****
references	[15]	[15]	[293]	[294]	[19]

**Fig. 2:**
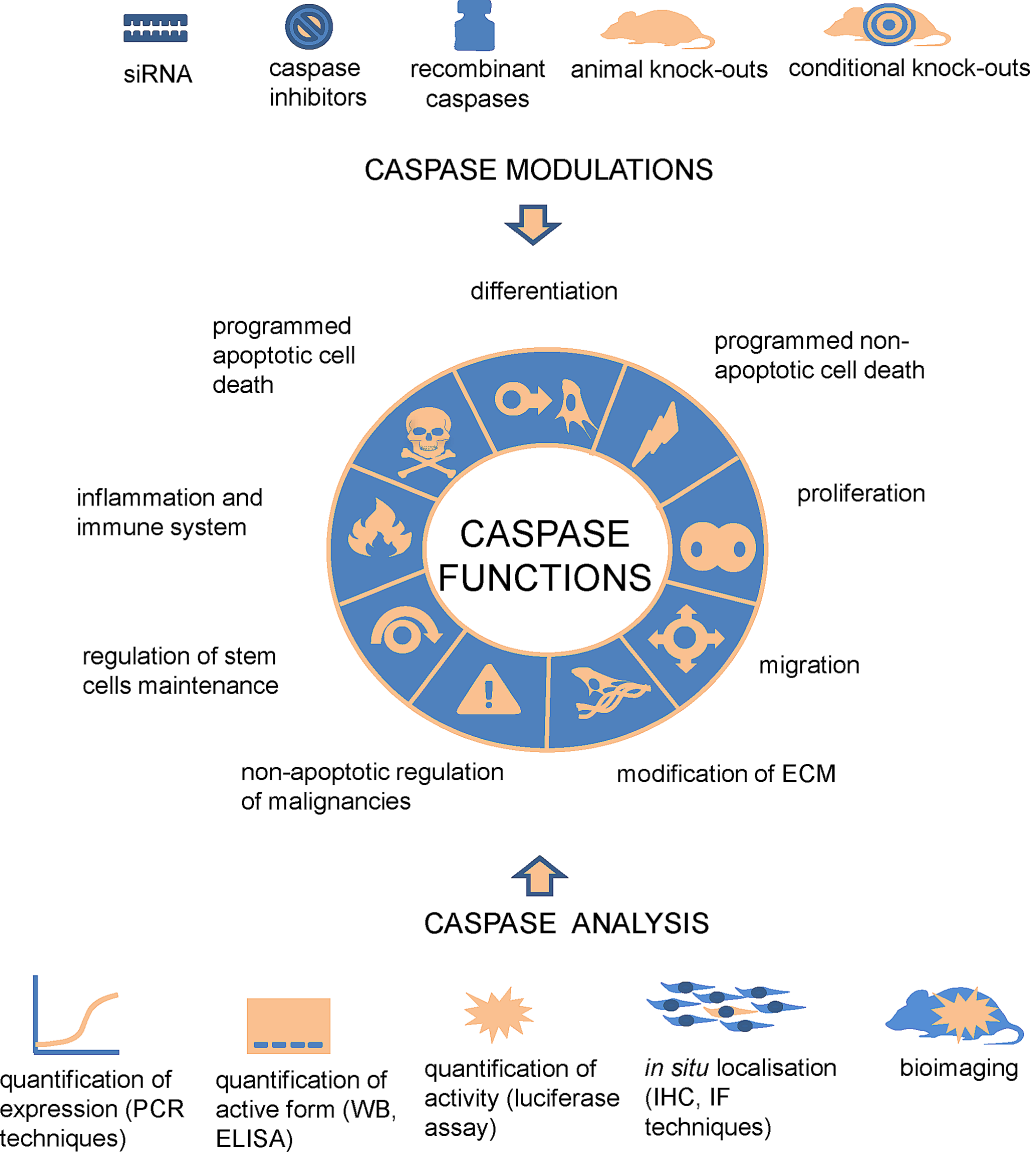
Overview of caspase modulation, analysis, and functions. Downstream caspase pathways may be studied with the help of the caspase downregulation at several levels, including inhibition of caspase gene expression by siRNA and inhibition of caspase activity by inhibitors in vitro. Alternatively, recombinant caspases may be used for specification of caspase functions. In vivo investigation relies on deficient mice with null or targeted caspase deletion. Analysis of caspases includes quantification of caspase expression by PCR-based techniques and activity assessment (e.g., western blot, bioluminescence, bioimaging) applied in vitro and in vivo. Detection of caspases by specific antibodies in situ provides information about caspase importance in individual cell types. With the help of these approaches, caspases have been associated with multiple functions such as programmed apoptotic cell death [286], programmed non-apoptotic cell death [287], inflammation and immune system [288], differentiation [16], proliferation [289], regulation of stem cells maintenance [43], non-apoptotic regulation of malignancies [290], modification of ECM [181], migration [28]

Caspase research was developed with help of various laboratory techniques as summarised in Fig. 2. Different organisms (Table 1) were used to investigate caspases and their downstream pathways, particularly, biological activities, potential redundancies, interactions, or impact/s of their deficiency. The mouse is the most relevant in vivo model to search for potential applications in several caspase-related human diseases such as autoimmune, inflammatory, cancer, metabolic, and neurodegenerative pathologies [17].

## Caspase structure and classification

**Fig. 3:**
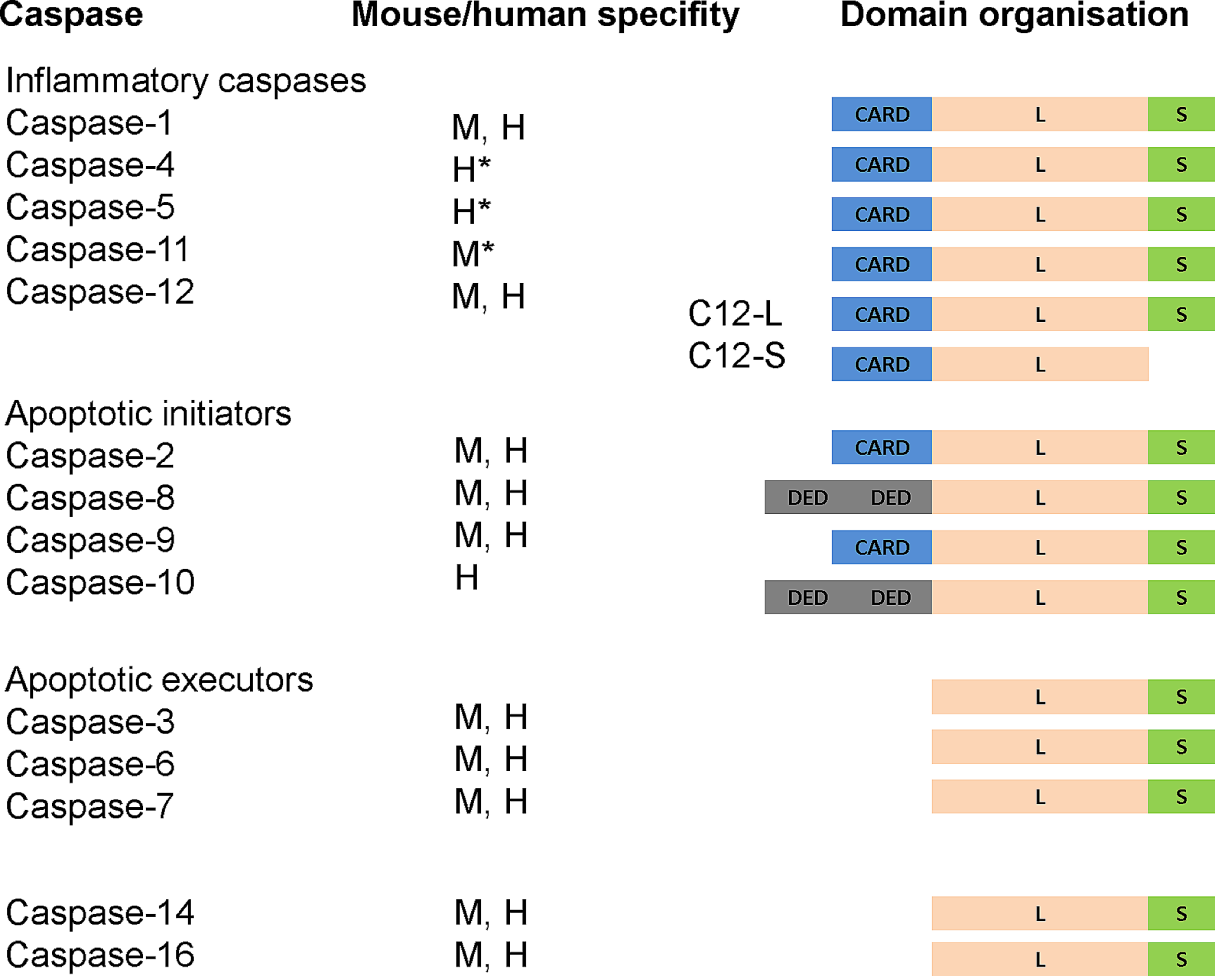
Classification of caspases. Blue/Grey: long pro-domain containing CARD/DED, pink: long domain L, green: short domain S. The asterisk is used to highlight human/mouse caspase orthologue caspase-4, caspase-5/caspase-11. C12L and C12S stands for full-length and truncated versions, respectively

Despite being known for dozens years, there is no clear order in caspase classification. Respectively, the recent classifications are rather artificial, and does not reflect all characteristic of individual caspases. We can also speculate that each caspase has both broad (redundant) and specific (non-redundant) functions. Therefore, any categorisation would inevitably be somewhat inaccurate. When evaluating lethal vs. non-lethal caspases (as shown in Fig. 3), caspase-2, -3, -6, -7, -8, -9, -10 are conventionally associated with apoptosis. Further, there is a group of inflammatory caspases with caspase-1, -4, -5, -11, -12. The mammalian caspases with unknown lethal function are caspase-14 [15, 18] and caspase-16 [10].

Pro-apoptotic caspases were further subdivided based on their molecular structure and the relation to the apoptotic machinery. Caspases are mostly expressed as inactive monomers consisting of a pro-domain (long or short), large, and small subunits. The long pro-domain is typical for initiator caspases. It may contain two death effector domains (DED), as seen in caspase-8 and -10. Alternatively, it can have a caspase-activation recruitment domain (CARD), found in caspase-2 and -9. These domains promote dimerization, followed by autoactivation in multiprotein complexes [19]. In contrast, executioner caspases lacking the long pro-domain require cleavage by initiator caspases to reach the activated state [20].

Regarding recent observations on caspase functions, some authors have proposed slight modification(s) to the original classification. In the new system, three caspases stand alone: caspase-2 as a caspase involved in cell cycle, caspase-14 as a caspase involved in cell differentiation, and caspase-12 as caspase with undefined functions [17].

The caspase family can also be subdivided according to amino acids making up the motif (P4, 3, 2, 1) upstream of the cleavage site P1 (Table 2) [21]. Several groups, using different methods, have demonstrated a problem of overlapping substrate specificity among caspases [22]. In the cleavage motif, there are positions where variations are tolerated compared to positions with high selectivity (Table 2). It is important to note that some caspases can cleave certain substrates better than others, sometimes unexpectly based on original analyses [23].

**Table 2:** Caspase cleavage motives - based on human caspase research according to Talanian et al. [21]. Caspases exhibit selectivity for Asp (D) in the P_1_ position, toleration of wide range of amino acids in the P2, a preference for Glu (E) in P_3_, and a lack of tolerance for charged residues at P_1_′ (*φ* symbol stands for preferred Gly, Ala, Thr, Ser and Asn). Most significant differences in caspase specificities are at the P4 positions. **↓** stands for the cleavage site. * Caspase-6 was recently identified to recognise and prefer pentapeptide motif [295]. These groups of caspase roughly reflect groups of initiators, executors, and inflammatory caspases

caspase	P5	P4	P3	P2	P1 ↓	P1´
-1, -4, -5, -14		W/Y	E	X	D	**Φ**
-8, -9, -10		I/L	E	X	D	**Φ**
-3, -7		D	E	X	D	**Φ**
-6 *		V	E	X	D	**Φ**
-2	V/L	D	E	X	D	**Φ**

## Caspase functions

Caspases have been first recognised as enzymes crucial for apoptotic cell death and inflammation. However, following studies pointed to their functions beyond lethal activities [15, 24–26]. These events include both “non-autonomous” and “autonomous” mechanisms. The former refers to mechanisms that mediate, for example, the compensatory proliferation of cells adjacent to those undergoing apoptosis, while the latter refers to intrinsically mediated activities of caspases that do not result in cell death [27]. Particularly, caspases were associated with proliferation [25], migration [28], differentiation of various cell types [29–32], or even inhibition of cell death [33]. Their substrates include proteins associated to various cellular functions not only the lethal ones but also substrates related to cell adhesion, cytoskeleton, physiology of endoplasmic reticulum (ER) and Golgi apparatus, cell cycle, DNA synthesis and repair, etc. [34]. The cleavage hit mediated by caspases may result in both activation [35] or inactivation [36] of the substrates.

Types and number of substrates is thought to be very different throughout caspase groups. Initiators are thought to cleave few substrates besides their own precursors and other caspases downstream, effectors have a broader spectrum of targets. Among executors, caspase-3 seems to be more promiscuous compared to caspase-7 [37, 38]. The fundamental roles of caspases are summarised in Fig. 2.

Diverse roles of caspases are thought to be associated with various molecular pathways. The apoptotic signalling of caspases is directed by so called extrinsic and intrinsic pathway [39, 40]. The extrinsic pathway regulated by interaction of death receptor (DR) and death ligand results in formation of death-inducing signalling complex (DISC) that activates initiator caspase-8, -10. The intrinsic (mitochondrial) pathway is triggered by internal signals inducing the leakage of cytochrome-c out of mitochondria. Cytochrome-c associates with Apaf-1 and pro-caspase-9, giving rise to a multiprotein complex known as apoptosome, where caspase-9 is activated [41].

Apoptotic pathways are modulated by diverse inhibitory apoptosis proteins (IAPs) and members of the B-cell lymphoma 2 (Bcl-2) protein family, which is divided into three groups: anti-apoptotic proteins (Bcl-2, Bcl-xl, Bcl-w, Mcl-1, Bfl-1/a1), pro-apoptotic BH3-only proteins (Bad, Bid, Bik, Bim, Bmf, Hrk, Noxa, Puma, etc.), and pro-apoptotic pore-formers (Bax, Bak, Bok) [42].

The extrinsic and intrinsic pathways are often interconnected and finally aim to activate central caspase-3 or other executors. In the apoptotic machinery, the executors are not equivalent in their capacity [38]. In short, caspase-3 engagement finally results in caspase-activated DNase (CAD) activation which causes degradation of nuclear DNA. Executors further play role in the cytoskeletal reorganization and formation of cytoplasmic blebs and apoptotic bodies.

**Fig. 4:**
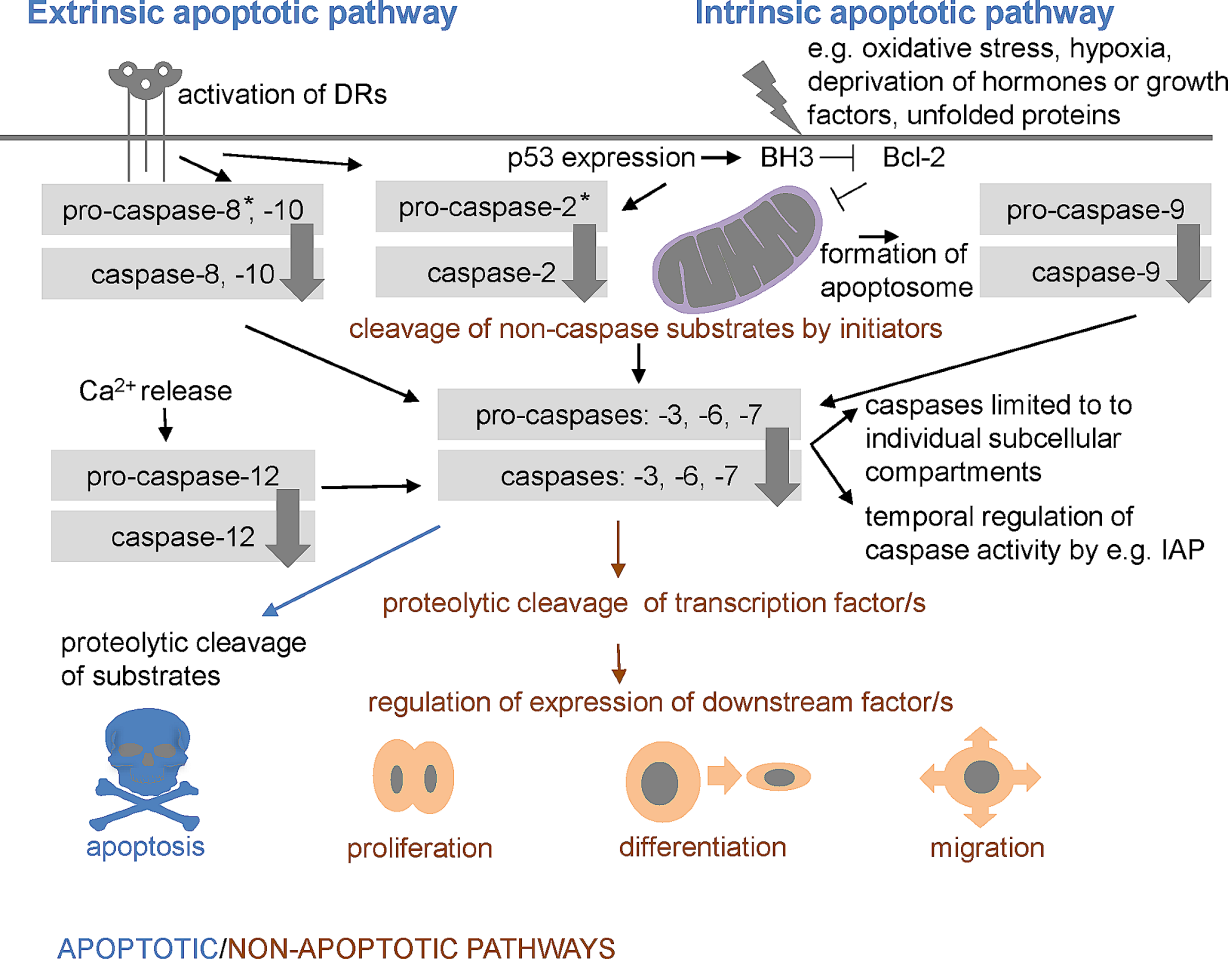
Apoptotic and non-apoptotic caspase signalisation. The extrinsic pathway is regulated by death receptors, leading to the activation of caspase-8 and -10. The intrinsic pathway is usually initiated in a cell-autonomous manner, resulting in expression of BH3-only proteins that inhibit anti-apoptotic proteins such as Bcl-2, permeabilization of the mitochondrial outer membrane, formation of apoptosome, and activation of caspase-9. Both pathways aim to activate caspase-3 (or other executors: caspase-6, -7). The extrinsic and intrinsic pathways are often interconnected (e.g. caspase − 8 (and also − 2) cleaves Bid into tBid, which impacts mitochondria). Caspase-12 contributes to Ca^2+^-dependent apoptosis. Caspase-2 activation occurs in response of both intrinsic and extrinsic stimuli

In contrast, non-lethal functions of caspases remain mostly unknown, although they may involve processes such as the cleavage of non-caspase substrates by initiators, signalisation of executor pro-caspases, or proteolytic cleavage of transcription factors [30, 43, 44] as illustrated in Fig. 4.

### Initiator caspases

The phenotypes of mice deficient for initiator caspases are listed in Table 3.

**Table 3:** Phenotypes of mice lacking initiator caspases. *Mutation in self processing site, d.p.c. day post coitum, HFSC hair follicle stem, NGF nerve growth factor

	mouse strain	development/ phenotype	apoptotic/ non-apoptotic/unspecified effect	reference
*Casp-2* ^*−/−*^	C57BL/6J	normal development	decreased number of oocytes, increased number of motoneurons	[46]
*Casp-2* ^*−/−*^	129/sv×C57BL/6	normal development	normal apoptosis of thymocytes induced by various stimuli, normal apoptosis of neurons in absence of NGF	[70]
*Casp-2* ^*−/−*^	C57BL/6J	reduced sensitivity to heat-shock	reduced cell death	[296]
*Casp-2* ^*−/−*^	C57Bl/6	premature ageing	compromised ability to clear oxidatively damaged cells	[67]
*Casp-2* ^*−/−*^ */MMTV*	129/B6	increased tumor acquisition	cell cycle defects, genomic instability	[75]
*Casp-2* ^*−/−*^	C57BL/6J	premature ageing	increased oxidative stress	[15]
*Casp-2* ^*−/−*^	C57BL/6J	age-related bone loss	increased number of osteoclasts due to reduced apoptosis and increased differentiation	[71, 72]
*Casp-2* ^*−/−*^	C57BL/6J	reduced white adipose mass, smaller white adipocytes etc.	altered balance in fuel choice towards increased carbohydrate utilisation due to mild energy stress	[76]
*Casp-8* ^*−/−*^	C57BL/6	abnormalities of heart etc.-prenatally lethal	abnormal receptor-dependent pathway of apoptosis	[68]
*Casp-8* ^*−/−*^ (conditional T-cells-specific)	C57BL/6	inability of immune response to viral infection	resistance of T-cells to death mediated by anti-CD95 antibody	[90]
*Casp-8*^*−/−*^(conditional-several types)	MF-1	lethal/ non-lethal phenotypes	apoptotic/non-apoptotic engagement in dependence of the targeted cells	[97]
*Casp-8* ^*−/−*^ (conditional B cells-specific)	-	altered responses of B cells to different stimuli	B cells: abrogation of Fas-mediated apoptosis, failure of induced proliferation, altered ab production	[91]
*Casp-8* ^*−/−*^ (conditional B cells-specific)	129/J × C57BL/6	normal cell subpopulations in bone marrow	resistance to DR mediated apoptosis, defective B cells expansion response to TLR4 stimulation, attenuated antibody production upon viral infection	[105]
*Casp-8* ^*−/−*^ (conditional keratinocytes-specific)	C57BL/6	inflammatory diseases of the skin-lethal by postnatal day 7	constitutive phosphorylation of interferon regulatory factor (irf) 3 and tank-binding	[94]
*Casp-8* ^*−/−*^ (conditional epidermis-specific)	-	epidermal hyperplasia	increased stem cell proliferation and cutaneous inflammation regulated by il-1α	[102]
*Casp-8* ^*−/−*^ (conditional hepatocytes-specific)	-	abnormal hepatocyte reaction to damage	apoptotic and non-apoptotic mechanisms of response to different stimuli	[106, 107]
*Casp-8* ^*−/−*^ (conditional macrophage-specific)	C57BL/6	mild systemic inflammatory disease	unchecked ripk3 activity	[101]
*Casp-8-/-* (conditional endothelium -specific)	-	reduced retina angiogenesis	reduced endothelial proliferation, sprouting, and migration, destabilization cadherin	[32]
*Casp-8*^*−/−*^D387A*	C57BL/6	normal development	reduced Fas-induced apoptosis of T cells, preservation of fas-mediated non-apoptotic signalling	[97]
*Casp-9* ^*−/−*^	129/C57BL/6 129/CD1	severe brain defect-prenatally lethal	resistance of different cells to apoptotic stimuli	[124]
*Casp-9* ^*−/−*^	C57BL/6	severe brain malformation- prenatally lethal	reduced apoptosis and activation of caspase-3 in brain, resistance of T cells to different apoptotic stimuli	[69]
*Casp-9* ^*−/−*^	C57BL/6	defects and size reduction of the inner ear	decrease of Apaf-1 dependent apoptosis	[116]
*Casp-9* ^*−/−*^	C57BL/6	misrouted axons	abnormal cleavage of sema7a	[122]
*Casp-9* ^*−/−*^	C57BL/6	increased number of oocytes	decreased apoptotic elimination of oocytes during a narrow window between 18.5 and 21.5 d.p.c.	[125]
*Casp-9* ^*−/−*^ (conditional HFSC-specific)	-	accelerated wound repair	induction of apoptotic-engaged state, serving as mitogenic signalling centres by releasing wnt3	[129]

#### Caspase-2

Caspase-2 is thought to be the most evolutionary conserved caspase [45] with a broad expression (brain, heart, kidney, lung and spleen) [46]. Based on the structural properties which include long pro-domain and dimerization during activation, caspase-2 is usually classified as initiator caspase [47]. However, according to research on substrate specificity, caspase-2 rather fits with executor group [21, 48, 49]. Due to the conflicting evidences of its activation (homodimerization, cleavage by other caspases, multiprotein complexes, etc.), function/s or signalling it is called an „orphan“ caspase [50–52]. Regarding apoptosis, caspase-2 can be either pro-apoptotic or anti-apoptotic depending on the cell type, state of growth, and apoptotic stimuli [46, 52]. Interestingly, two splice-variants of caspase-2, pro-apoptotic caspase-2 L, and anti-apoptotic caspase-2 S (included also in DNA repair) were identified to be generated from the same gene in response to pro-apoptotic stimuli [7, 53]. Caspase-2 was also suggested to induce lipoapoptosis, cell death triggered by excessive intracellular accumulation of long-chain fatty acids [54]. Caspase-2-deficient mice did not manifest a phenotype that would support a broad function for caspase-2 in apoptosis [46]. In contrast, this caspase exhibits numerous non-lethal functions, serving as a tumour suppressor [55]/ a cell cycle regulator [52], a regulator of genomic integrity [56, 57], and participating in various cellular processes such as the differentiation [58] and protection of neurons [46], the differentiation of skeletal muscles [59, 60], and osteoblasts in vitro [61, 62]. It might also serve as a therapeutic target for neurological diseases [63], non-alcoholic steatohepatitis [64], metabolic syndrome [54], tautopathies [65], cancer [66], and factor impacting aging [67]. Caspase-2-deficient mice are viable with no gross anatomic abnormalities [46].

#### Apoptotic effect of caspase-2 deficiency

Caspase-2-deficient mice [46] do not suffer from severe developmental abnormalities, as documented in the case of other initiators [68, 69], implying its crucial function extends beyond apoptosis. Based on caspase-2-deficient mice, it is not clear whether caspase-2 is pro- or anti- apoptotic. Caspase-2 was proposed to stimulate apoptosis of primordial follicles [46]. The number of newly formed primordial follicles containing oocytes was significantly higher in caspase-2-deficient females when compared with wild type (WT) mice, suggesting that apoptotic elimination of foetal germ cell was attenuated in the absence of caspase-2. Furthermore, the oocytes were found to be resistant to cell death induced by chemotherapeutic drugs. This phenomenon, however, was strictly associated with oocytes. Other cell types, such as thymocytes and dorsal root ganglion (DRG) neurons, did not show alterations in apoptotic cell death [70]. In contrast to this, caspase-2 protected motor neurons against naturally occurring cell death during embryonic development, since new-born mice with caspase-2 deficiency had decreased number of motor neurons. The phenomenon might be explained by expression rate of caspase-2 L/caspase-2 S. The short isoform appears to be present in terminally differentiated tissues, such as brain, where it may play a role in survival. Alternatively, caspase-2 loss might be compensated by other caspases up-regulating their expression [46].

#### Non-apoptotic effect of caspase-2 deficiency

The signs of caspase-2-deficient mice, apart from cell death, indicate a broad functional complexity of this enzyme. While caspase-2-deficient mice had almost the same median lifespan as WT mice, they statistically lived shorter lives [67]. Interestingly, caspase-2 deficiency promoted a number of traits commonly seen in aging animals [15, 67], making these mice potentially interesting models for age-related diseases. Some age-related outcomes may result from significantly increased oxidative damages and reduced activity of antioxidant enzymes in old caspase-2-deficient mice compared to WT animals. The underlying mechanism may include reduced expression of FoxO transcription factors and increased levels of p53 and p21 [16]. The oxidative stress was associated with lower bone mineral density in old (24–26 months) caspase-2-deficient mice compared to WT mice, potentially increasing osteoclast differentiation and reducing apoptosis, leading to enhanced bone resorption [71, 72]. Additionally, lower body fat content and impaired hair growth in caspase-2 deficient mice [67] may be related to oxidative damage.

In the context of neurodegenerative diseases associated with advanced age, mice deficient for caspase-2 showed rescued behavioural and cognitive features of Huntington’s disease (HD) in the YAC128 model. However, they did not exhibit protection from anatomical abnormalities associated with HD, such as specific striatal volume loss [73]. This suggests that different pathways may be involved in the behavioural changes observed in HD. Inhibition of caspase-2 activity could potentially be associated with symptomatic improvement in HD.

Disruption of p53 regulated pathway found in caspase-2-deficient mice may be also related to higher tumour incidence at a sooner age as was seen in caspase-2^−/−^/MMTV model. Mechanism of caspase-2 action might reside in regulation of cell cycle progression and genomic stability [45, 55, 74, 75]. However, the overall tumour incidence was not observed in caspase-2 deficient mice [67]. Therefore, caspase-2 may be specifically involved in the process of carcinogenesis.

Besides age-related abnormalities, caspase-2 deficiency also altered basal energy metabolism by shifting the balance in fuel choice from fatty acid to carbohydrate usage. Four weeks old caspase-2-deficient mice had increased carbohydrate utilisation and by 17 weeks showed a reduced white adipose mass, smaller white adipocytes, decreased fasting blood glucose and plasma triglycerides but maintained normal insulin levels. In addition, caspase-2-deficient mice placed on a high-fat diet resisted the development of obesity, fatty liver, hyperinsulinemia, and insulin resistance [76].

#### Caspase-8

Caspase-8 was described as the major initiator of the extrinsic apoptotic pathway [8, 9, 77]. Caspase-8 was identified in cytoplasm as an inactive dimer activated by self-processing [78], which is induced *via* interaction of the DRs with their ligands. Apart from the apoptosis, caspase-8 is essential for inhibition of necroptosis mediated by Receptor Interacting Serine/Threonine Kinase (RIPK3) and Mixed Lineage Kinase domain-Like (MLKL) [79–82]. Further, it is involved in pyroptosis [83], inflammation [84–86], migration [87, 88], cellular proliferation [89–92], differentiation of osteoblasts [61, 62], myoblasts [29], autophagy [93], and overall cell homeostasis [94]. Caspase-8 is thought to be potential target for treatment of oncologic [95], inflammatory or immune pathologies [96]. Caspase-8-deficient mice performed prenatal lethality around the stage E12.5 resulting from gross abnormalities of vasculature and yolk sac [68].

#### Apoptotic effect of caspase-8 deficiency

The role of caspase-8 in apoptosis was identified in mesenchymal embryonic fibroblasts (MEF) derived from caspase-8-deficient mice that developed a resistance to extrinsic pathway of programmed cell death [68]. Apoptotic role of this caspase has been further demonstrated in vivo, when caspase-8 specific deletion in hepatocytes using Cre/loxP system protected these cells from Fas-mediated cytotoxicity [97].

#### Non-apoptotic effect of caspase-8 deficiency

Despite being classified as an apoptotic activator, caspase-8 is vitally important for its anti-lethal effect as a regulator of necroptosis. Interestingly, caspase-8 also appears to regulate inflammatory processes, likely stemming from complex molecular pathways that are not yet fully understood. Deletion of caspase-8 in mice revealed the huge impact of this molecule/protease on the murine embryonic development. The deficiency resulted in degeneration of yolk sac and its vasculature leading to hyperaemia of some blood vessels and organs, congested accumulation of erythrocytes, impaired heart muscle development and neural tube defects [68, 97, 98]. Due to early lethality of caspase-8-deficient mice, following studies focused on targeted deletion of caspase-8 in specific cell populations. Later research explained that lethality of caspase-8-deficient embryos was consequence of an abnormal activity of RIPK3 which is the key component of the necrosome [99]. Caspase-8 inhibits RIPK3 and thus prevents engagement of the final effector MLKL triggering necroptosis [80]. Deletion of RIPK3 or MLKL rescued the embryonic lethality of caspase-8-deficient mice [80, 82]. MLKL deficiency rescued the cardiovascular phenotype but unexpectedly caused perinatal lethality in mice with catalytically inactive caspase-8 (Casp8C362S/C362S,) indicating that CASP8 (C362S) causes necroptosis-independent death at later stages of embryonic development [33].

Despite mice lacking both caspase-8 and RIPK3 not showing any histological abnormalities *in utero*, embryonic upregulation of the inflammatory genes was detected in several tissues. Interestingly, when focused on the liver, the expression of inflammatory genes starts preferentially in endothelial cells, which were also primarily impacted in caspase-8-deficient mice with fatal consequences [100]. In contrast to increased inflammatory expression in mice lacking both caspase-8 and RIPK3, the loss of caspase-8 in macrophages promotes the onset of a mild systemic inflammatory disease, which could be prevented by the deletion of RIPK3 [101]. Therefore, cell-specific mechanisms probably exist. Regarding the inflammatory processes, mice producing enzymatically inactive caspase-8 developed an inflammatory disease of skin associated with a hyperproliferative state [94], resulting from abnormal signalling regulated by IL1α, which activates both stem cell proliferation and inflammation [102]. Inflammation was also detected in the intestines of mice with conditional deletion of caspase-8 [32].

Caspase-8 deletion further resulted in cellular/humoral alterations of the immune system. Mice with targeted deletion of caspase-8 had significant decrease in the number of peripheral T-cells that were unable to mediate an immune response to viral infection [90]. Notably, in the same mice but older, B and T cell compartments were expanded in the absence of any infection, which resulted in lymphoproliferation and a lethal T cell infiltrating disorder [103]. The impaired function of T-cells was associated with modulation of nuclear factor κB (NF-κB), a key transcription factor for activation of T-cells [104]. NF-κB was also linked with decreased production of antibodies and impaired survival following stimulation of the Toll-like receptors of B-cells in mice with B-cell-specific inactivation of caspase-8 [105].

Generation of mice lacking caspase-8 in hepatocytes (caspase-8^Δhepa^) demonstrated the role of caspase-8 in liver regeneration after partial hepatectomy. The loss of caspase-8 prevented proteolytic cleavage of the receptor-interacting protein 1 (RIP1) in hepatocytes and subsequently triggered premature activation of NF-κB and c-Jun N-terminal kinase (JNK) related signals which leads to improved liver regeneration [106, 107].

#### Caspase-9

Caspase-9 is an initiator of the intrinsic apoptotic pathway that becomes activated in apoptosome. Alternatively, activation of caspase-9 without Apaf-1 was induced is some cells by insulin deprivation [108] or caspase-9 can be even cleaved by caspase-3 [109]. In contrast to other caspases, pro-caspase-9 manifests a basal activity that increases with activation level [110]. During apoptosis, caspase-9 cleaves effector caspases [111] or non-caspase substrates (such as vimentin) [111] to dismantle intermediate filaments and amplify the cell death signal [112]. Notably, caspase-9 may also negatively regulate apoptosis with alternatively-spliced truncated caspase-9b form competing with full length caspase-9 [113]. Developmental importance of caspase-9 is supported by its early activation in mouse embryo and early lethality resulting from severe developmental defects [114–116]. Caspase-9 also contributes to necroptosis [117]. Further, caspase-9 was associated with non-lethal functions [118] such as myocyte differentiation and proliferation [119], hematopoietic development [120], immune response to viral infection [121], axon guidance [122] or axon-selective degeneration [44] etc. In a therapeutic invention, caspase-9 may play a central role in pathogenesis of stroke, neurodegenerative diseases, or brain injury caused by hypoxia [123]. Caspase-9 deletion is embryonically or perinatally lethal due to aberrant brain development [69, 124].

#### Apoptotic effect of caspase-9 deficiency

The most apparent abnormalities of caspase-9 deficiency resided in large brain protrusions and other defects mostly localised in the cortex and forebrain [124]. These alterations were associated with decreased rate of apoptosis and excessive number of neurons. Caspase-9 deficiency further resulted in dramatic decrease in apoptosis in the inner ear epithelium, severe morphogenetic defects, and a significant size reduction of the membranous labyrinth [116].

Furthermore, several cell types performed an abnormal apoptosis when challenged by different apoptotic stimuli. This was, however, not seen in TNF-α induced apoptosis in MEF of caspase-9-deficient mice [124].

Caspase-9 was shown to play a role in oocyte elimination during development. In caspase-9-deficient mice, later phase of oocyte loss was prevented and the total number of oocytes became significantly greater in caspase-9-deficient ovaries at E19.5 when compared to normal mice [125].

In the prenatal formation of tooth, caspase-9-deficient mice displayed inhibition of apoptotic cell death in the primary enamel knot (PEK) [126], the signalling centre of the first molar [127]. Despite PEK regulates the bud-cap transition [128], no impact of the decreased apoptosis was observed during advanced tooth development, indicating that the apoptotic cell death mediated by caspase-9 has been compensated by other molecular mechanisms [126].

Caspase-9 deletion in hair follicle stem cells attenuated the apoptotic process, which surprisingly resulted in increased levels of cleaved caspase-3. These cells were retained in an apoptotic-engaged state, serving as mitogenic signalling centres by releasing Wnt3. Notably, these mice displayed accelerated wound repair and *de novo* hair follicle regeneration [129].

#### Non-apoptotic effect of caspase-9 deficiency

Caspase-9 was identified as being important for non-apoptotic aspect(s) of neural development, such as axon-selective degeneration. Interestingly, Apaf-1 was not essential for the process, suggesting either Apaf-1 independent caspase-9 activation [44] or dependence of the phenomenon on pro-caspase form.

Staying with neural system, caspase-9-deficient mice exhibited misrouted axons, impaired synaptic formation, and defects in the maturation of olfactory sensory neurons without affecting the number of these cells. Caspase-9 was shown to be engaged in regulation of active Sema7A levels, which affects axonal path finding, synapse formation and maturation status in the olfactory bulb [122].

### Executor caspases

The phenotypes of mice deficient for executor caspases are listed in Table 4.

**Table 4:** Phenotypes of mice lacking execution caspase

	mouse strain	development/ phenotype	apoptotic/ non-apoptotic/unspecified effect	reference
*Casp-3* ^*−/−*^	129 × 1/SvJ	severe brain defects-perinatally lethal	decreased apoptosis of neural precursors	[131]
*Casp-3* ^*−/−*^	C57BL/6J	decreased body size, ectopic masses in head	reduced apoptosis in diverse settings	[148]
*Casp-3* ^*−/−*^	C57BL/6	progressing deafness	degeneration of spiral ganglion neurons and a loss of inner and outer hair cells	[153]
*Casp-3* ^*−/−*^	C57BL/6	abnormal development of inner ear	putative defective apoptosis	[144]
*Casp-3* ^*−/−*^	C57BL/6J	minimal brain defect	slight resistance to induced cell death	[141]
*Casp-3* ^*−/−*^	B6.129S1	reduction of skeletal muscle mass	defective myoblast differentiation, reduced activation of mst1	[29]
*Casp-3* ^*−/−*^	C57BL/6J	increased number of B cells	increased proliferation of b cells (increased cdk activity and cyclin abundance)	[134]
*Casp-3* ^*−/−*^	B6.129S1	delayed ossification, decreased bone mineral density	over activation of tgf-b/smad2 pathway, upregulation of p53 and p21, downregulation of cdk2 and cdc2	[30]
*Casp-3* ^*−/−*^	C57BL/6J 129 × 1/SvJ	slight alterations of molar development	absence of apoptotic bodies in molar tooth germ at E15	[145]
*Casp-3* ^*−/−*^	C57Bl/6	increased immature hematopoietic cells	impact on hematopoietic stem cell homeostasis	[149]
*Casp-3* ^*−/−*^	C57Bl/6	decreased skin wound healing and liver regeneration	abnormal pge2 production	[189]
*Casp-3* ^*−/−*^	C57BL/6J	small body size at birth, inner ear abnormalities	putative apoptotic mechanisms	[133]
*Casp-3* ^*−/−*^	C57Bl/6	decreased incidence of induced skin cancer	attenuation of endog	[147]
*Casp-3* ^*−/−*^	B6.129S1	reduced sebaceous glands	downregulation of yap and genes of proliferation	[150]
*Casp-3* ^*−/−*^	C57Bl/6	proliferative glomerular lesions, splenomegaly	expression of inflammation-associated genes	[146]
*Casp-3* ^*−/−*^	C57BL/6	ADHD, signs of autism in males	*putative disruption of homeostatic synaptic plasticity*	[154, 155]
*Casp-3* ^*−/−*^ *7* ^*−/−*^	C57BL/6	perinatal lethality	exencephaly in 10% of embryos, heart abnormalities	[186]
*Casp-3* ^*−/−*^ *7* ^*−/−*^ (conditional myocardium-specific)	C57BL/6J	hypoplastic heart at birth, myocyte hypertrophy	reduction of myocyte proliferation, increase of glycolytic enzymes	[297]
*Casp-6* ^*−/−*^	C57BL/6J	increased susceptibility to influenza infection	altered cell death, zbp1-mediated inflammasome activation, and host defense	[298]
*Casp-6* ^*−/−*^	C57BL/6	abnormal development of B cells	no difference of apoptosis b cells activation and differentiation of plasma cells	[167]
*Casp-6* ^*−/−*^	C57BL/6	protection of neurons against stroke	reduced loss of processes and soma of neurons	[168]
*Casp-6* ^*−/−*^	FVB/NJ	neuroanatomical and behavioural alterations	protection from excitotoxicity, ngf deprivation and myelin-induced axonal degeneration	[163]
*Casp-6* ^*−/−*^	C57BL/6J	alterations in B cells subsets	alteration of il-7 mediated signalling	[172]
*Casp-6* ^*−/−*^	C57	attenuated liver damage in response to I/R	altered regulation of nr4a1/sox9 interaction	[169]
*Casp-7* ^*−/−*^	C57BL/6	normal development	slight survival advantage of MEF after cell death induction	[186]
*Casp-7* ^*−/−*^	C57BL/6	protection from LPS-induced lethality	resistance to LPS-induced lymphocyte apoptosis	[177]
*Casp-7* ^*−/−*^	C57BL/6	abnormal development of hard tissues	alteration of gene expression associated with formation of hard tissues	[31, 179]
*Casp-7* ^*−/−*^	C57BL/6	protection against ON injury-induced RGC loss	increased density of RGCs, reduced thinning of retina resulting from reduced cell death	[187]
*Casp-7* ^*−/−*^	C57BL/6	increased population of mast cells	putative abnormalities of non-apoptotic signalling	[299]

#### Caspase-3

Caspase-3 is widely expressed central executor caspase [1, 37]. In vitro investigation of caspase substrates highlighted caspase-3 as promiscuous enzyme with large spectrum of substrates [23]. Due to its central role, variable levels of caspase-3 are ubiquitously expressed in normal tissues [27]. Caspase-3 activation is mediated by both receptor and mitochondrial apoptotic signalling pathways. Additionally, a shorter isoform, caspase-3s, generated by alternative splicing, negatively regulates apoptosis [130]. Beyond crucial function of caspase-3 in apoptotic cell death during development [131–133], caspase-3 was associated with many non-apoptotic events such as regulation of cell cycle [134], cell differentiation [29, 30, 61, 135], stem cell physiology [43], tissue regeneration, and immunomodulation [136, 137]. Caspase-3 is considered as potential target for immunotherapy in distinct tumours [138], neurodegenerative disorders [139], or heart failure [140]. The phenotype of caspase-3-deficient mice was strain-specific. Caspase-3-deficient 129 × 1/SvJ mice died during the perinatal period and exhibited decreased programmed cell death in brain regions resulting in significant neural precursor cell expansion and exencephaly, ectopic, and duplicated neuronal structures. In contrast, caspase-3-deficient C57BL/6J mice reached adulthood, were fertile, and exhibited minimal brain pathology [141].

#### Apoptotic effect of caspase-3 deficiency

Caspase-3 mediated apoptosis was found to be indispensable for normal development of central nervous system [142] in caspase-3-deficient 129 × 1/SvJ mice. Compensatory activation of other caspase effectors in the caspase-3-deficient C57BL/6J, but not 129 × 1/SvJ, could be explanation for the strain-dependent phenotypes. And indeed, increased activation of caspase-7 was detected in C57BL/6J caspase-3-deficient mice [143]. Alternatively, strain-specific endogenous inhibitors of apoptosis may underlie the variable caspase-3-deficient phenotype [141].

In contrast to the increased mass of neural tissue, caspase-3-deficient eyes were smaller than their WT counterparts. Additionally, caspase-3-deficient mice displayed peripapillary retinal dysplasia, delayed regression of vitreal vasculature, and retarded apoptotic kinetics of the inner nuclear layer. It was assumed that this phenotype is a result of delayed apoptosis in the developing eye [132]. Therefore, in this case, caspase-3-related apoptosis may be more likely to be of regulatory importance (e.g. regulation of number of specific molecular signals-emitting cells) than basically elimination of unwanted cells. Abnormal organ “sculpturing” in caspase-3 deficiency was also case of the inner ear. Caspase-3 knockout mice developed hypomorphism of the vestibular organs resulting in abnormal locomotion and circling behaviour of mice [133]. Other study also pointed to hyperplasia of supporting cells and degeneration of sensory cells resulting in the hearing loss in caspase-3-deficient mice [144]. The role in “sculpting process” would be expected also for apoptosis of developing molar PEK, where caspase-3 was identified. Surprisingly, the absence of caspase-3 on the B57BL/6 background only led to disorganized epithelium of the developing tooth germ [145].

Despite almost normal life span of caspase-3-deficient mice with B57BL/6 background, they also suffered from some defects associated with abnormal apoptosis. These knock-out mice had kidney proliferative glomerular lesions characterized by increased cells and expression of inflammation-associated genes, but renal dysfunction was not observed. Furthermore, these mice had mild splenomegaly compared with WT mice [146].

Mice deficient in caspase-3 performed reduced chemically induced skin carcinogenesis. Thus, caspase-3 seems to facilitate, rather than suppresses, chemical-induced genetic instability and carcinogenesis. This contrasts with typically considered anti-oncogenic role of caspase activation, which ensures the elimination of genetically unstable or damaged cells [147].

#### Non-apoptotic effect of caspase-3 deficiency

Several studies have described a smaller body size in caspase-3-deficient mice compared to WT mice of the same age [30, 131, 148, 149]. One explanation for this phenomenon could be decreased cell proliferation. Indeed, decreased proliferation potential was identified in bone marrow stromal stem cells [30]. Deletion of caspase-3 further resulted in reduced cell proliferation, decreased cell number, and reduced sebaceous gland size. The underlying mechanism involved caspase-3-mediated cleavage of α-catenin, which facilitated the activation and nuclear translocation of yes-associated protein (YAP). YAP promotes the transcription of genes associated with cell proliferation [150]. Proliferation defects were also identified in hematopoietic cells, as caspase-3 alters signal transduction by limiting activation of the Ras-Raf-MEK-ERK [149], among others, thereby impacting proliferation.

Caspase-3 deletion resulted also in abnormal cell differentiation as was proved in different cell types. Impaired osteoblastic and osteoclastic differentiation was detected in caspase-3-deficient mice. Regarding the molecular signals, over-activated TGF-β/Smad2 pathway, which may lead to the compromised Runx2/Cbfa1 expression, was detected in preosteoblasts. Furthermore, the upregulated expression of p53 and p21, along with downregulated expressions of Cdk2 and Cdc2, and ultimately increased replicative senescence, were identified in caspase-3-deficient mice. These alterations ultimately resulted in delayed ossification and decreased bone mineral density in caspase-3-deficient mice compared to WT mice [30]. The role of caspase-3 in osteoclast differentiation was later confirmed, with primary osteoclasts unable to differentiate in response to RANKL in the absence of pro-caspase-3 [151]. Furthermore, caspase-3 deficiency impacted the differentiation of myoblasts, leading to a total reduction in skeletal muscle mass. This effect was associated with proteolytic function of caspase-3 that activates pro-myogenic Mammalian Sterile Twenty-like kinase (MST1) [29].

Since the process of regeneration includes both proliferation and differentiation, making it unsurprising that mice lacking caspase-3 exhibited deficiencies in skin wound healing and in liver regeneration [152]. Furthermore, the complexity of caspase-3 functions extends to its impact on hematopoietic stem cells homeostasis detected in caspase-3-deficient mice [149].

Caspase-3 seems to be important for cell survival ganglion cells and hair cells. Caspase-3 knockout mice developed deafness with accompanying degeneration of spiral ganglion neurons and hair cells in the inner ear. The ganglion neurons in caspase-3 exhibit morphological features characteristic for necrosis [153]. Neural development of caspase-3-deficient mice was associated with behavioural changes similar to symptoms of attention deficit/hyperactivity disorder (ADHD) or autism-like social interactions [154]. The mechanism of such caspase engagement is poorly understood, however, in vitro results suggest a role of caspase-3 in expression of AMPA receptors mediating synaptic transition [155].

#### Caspase-6

Caspase-6 structure is similar to other executor caspases [156]. However, its contribution to apoptotic machinery is probably limited or peculiar [157, 158]. Furthermore, the substrate specificity of caspase-6 more closely resembles that of the initiator caspases, caspase-8 and caspase-9 rather than the two executioners, caspase-3 and caspase-7 [156]. In addition to activation by initiators, caspase-6 may be activated by caspase-3 [38]. Additionally, caspase-6 can act downstream of caspase-1 [159]. Caspase-6 participates in inflammasome activation and host defence mechanisms [160, 161]. Recently, it has also been linked with PANoptosis, a process that involves pyroptosis, apoptosis, and necrosis in the context of cancer pathologies [162]. Caspase-6 is extensively expressed in the brain and is associated to neurological disorders such as Alzheimer disease (AD) and HD, where it also seems to have therapeutic potential [163–165]. Gross developmental defects have not been identified in caspase-6-deficient mice [166–168].

#### Apoptotic effect of caspase-6 deficiency

Only a few apoptotic functions were described in caspase-6-deficient mice. Caspase-6 was associated with participation in ischemia/reperfusion (I/R) injury [169]. The engagement of caspase-6 in programmed cell death was also observed in caspase-6-deficient macrophages infected with influenza A virus (IAV). Impact of caspase-6 deficiency on apoptosis was manifested by attenuated cleavage of initiator caspase-8 and executioner caspase-3 and -7 [160].

#### Non-apoptotic effect of caspase-6 deficiency

Caspase-6 seems to play a significant role in neurodegeneration and the modulation of immune response. Caspase-6-deficient mice have shown protection from axonal degeneration, leading to improved in functional outcomes during ischemia [168]. However, the impact of caspase-6 on the neural system extends further, as evidenced by age-dependent behavioural changes and region-specific neuroanatomical alterations. These include increases in cortical and striatal volume accompanied by hypoactive phenotype and learning deficits observed in caspase-6-deficient mice [163]. Some of these abnormalities bear resemblance to the morphological or behavioural pathologies of AD and HD, which result from axonal degeneration [163]. The mechanism might be mediated by cleavage of β-amyloid precursor protein (APP) by beta-secretase during trophic factor deprivation. APP binds to DR6 leading to degeneration of axons by caspase-6 [170].

Caspase-6 was revealed in host defence against IAV infection and loss of caspase-6 impaired viral clearance. Reduced Z-DNA-binding protein 1 (ZBP1)-mediated NOD-, LRR- and pyrin domain-containing protein 3 (NLRP3) inflammasome activation was observed in caspase-6-deficient bone marrow-derived macrophages [160]. NLRP3 is known as an intracellular sensor that detects a broad range of microbial motifs and mediates formation of NLRP3 inflammasome leading to activation of caspase-1 and release of cytokines [171].

Caspase-6 was further observed to control the balance between cell proliferation and differentiation by cleaving substrates involved in maintaining B cell quiescence [167]. Increased number of G_1_ cells in caspase-6-deficient mice did not translate into dysregulation of overall B cell numbers in adult mice, but rather into an elevation of serum immunoglobulin levels [172].

#### Caspase 7

Caspase-7 was described as an executor of apoptosis [143, 173, 174], functionally distinct from caspase-3 [173], with a recently discovered non-canonical function as death facilitator [175]. Moreover, it was observed to participate in inflammation [176, 177]. Caspase-7 activation during apoptosis is mediated *via* initiator caspases. Under inflammatory conditions, caspase-7 activation requires caspase-1 inflammasomes [176, 178]. Additionally, various non-lethal functions were further associated with caspase-7, such as bone formation [179], mineralisation of incisor enamel [31], regulation of mast cell population in dermis [180], or modulation of extracellular matrix in vessels [181]. Caspase-7 inhibition has potential application in neurodegenerative disorders such as AD and HD [182] and prevention of lymphocyte cell death in sepsis [183]. Caspase-7 gene has been linked with rheumatoid arthritis [184], and insulin-dependent diabetes mellitus [185]. Caspase-7-deficient mice are born with normal appearance, organ morphology, and lymphoid development [186].

#### Apoptotic effect of caspase-7 deficiency

Since the caspase-7-deficient mice are mostly normal, it is not easy to judge whether its role in distinct organ systems is very specific, involves fine tuning, or if caspase-7 functions are compensated by other enzymes. In accordance with this, caspase-7-deficient MEFs only exhibited a slight survival advantage as compared with normal MEFs when treated with inducers of apoptosis. The authors of the study speculate about compensation by caspase-3 [186].

In contrast, caspase-7-deficient mice were protected against lipopolysaccharides (LPS)-induced mortality and LPS-induced lymphocyte apoptosis, independently of the excessive production of serum cytokines, showing that caspase-7 is not required for the secretion of pro-inflammatory cytokines and chemokines in this process [177]. Further studies on optic nerve (ON) injury indicated a significant apoptotic role of caspase-7 in the process. Optic nerve crush caused a progressive loss of retinal ganglion cells (RGCs), which was reduced in caspase-7-deficient mice. ON-induced thinning of ganglion cell complex was significantly ameliorated in caspase-7-deficient mice after injury as well [187].

#### Non-apoptotic effect of caspase-7 deficiency

Caspase-7 deficiency coincided with an altered expression of osteogenic markers, proposing a role of caspase-7 in differentiation of bone cells. Diverse effects were detected in intramembranous vs. endochondral bones. Intramembranous caspase-7-deficient bone showed a statistically significant decrease in volume while mineral density was not altered. Conversely, endochondral bone showed constant volume but a significant decrease in mineral density in the mutant mice [179]. This might point to multiple downstream functions of caspase-7, which are selectively applied in the two models of ossification.

Caspase-7 deficiency further resulted in delayed mineralization and/or hypomineralization of incisor enamel [31]. Notably, caspase-7 has a different localisation in the epithelial cells on the lingual side of rodent incisor where enamel is not secreted (caspase-7 negative) and the labial side of continuously renewing ameloblasts (caspase-7 positive). It is possible that caspase-7 is involved in the modulation of ameloblast functional differentiation by cleaving its direct target Oct4 [188], which was located in the cervical loop, a stem cells niche where progenitors of future ameloblasts reside [189].

Caspase-7 was speculated to regulate the number of mast cells localised in the dermis [180]. Notably, cleaved caspase-7 was observed in mast cells and its deficiency in adult skin resulted in an increased mast cell number.

### Inflammatory caspases

The phenotypes of mice deficient for inflammatory caspases are listed in Table 5.

**Table 5:** Phenotypes of mice lacking inflammatory caspases. *Mutant mice contain transgenic caspase-11, since caspase-1 and -11 are too close in the genome to be segregated by recombination. Consequently, caspase 1^–/–^ mice lack both caspase-11 and caspase-1. HS haemorrhagic shock

	mouse strain	development/ phenotype	apoptotic/ non-apoptotic/unspecified effect	reference
*Casp-1* ^*−/−*^	C57BL/6J	normal development	thymocytes resistant to FasL apoptosis, abnormal il-1 distribution	[201]
*Casp-1* ^*−/−*^	129/Sv	normal development	no alterations in apoptosis, defect in il-1 production	[202]
*Casp-1* ^*−/−*^	C57BL/6J	decreased brain damage	*reduced edema and lesions caused by ischemic injury*	[217]
*Casp-1* ^*−/−*^	C57BL/6J	protection against ARF	*various effects*	[198, 214]
*Casp-1* ^*−/−*^	C57BL/6J	prolonged response of lungs to LPS	altered regulation of apoptosis, absent il-1β production in neutrophils treated by lps	[205]
*Casp-1* ^*−/−*^	C57BL/6J	abnormal reactions to bacterial infection	*diverse effects*	[210, 211]
*Casp-1* ^*−/−*^	C57BL/6J	protection against cisplatin-induced ATN	protection from apoptosis	[216]
*Casp-1* ^*−/−*^	C57BL/6J	thicker retina after light exposure and I/R injury	apoptosis of retinal neurons after excessive light exposure and I/R injury	[206]
*Casp-1* ^*−/−*^	-	improved myocardial infarction	reduced apoptosis associated with decreased activation of caspase-3	[204]
*Casp-1* ^*−/−*^	C57BL/6J	increased susceptibility to IAV	decreased cytokine production	[209]
*Casp-1* ^*−/−*^	C57BL/6J	increased susceptibility to induced tumorigenesis	reduced apoptosis, increased proliferation	[207]
*Casp1* ^*−/−*^ Casp11Tg*	C57BL/6	altered response to bacterial infection	failure in secretion of il-1b and il-18 in response to various stimuli, lethal response to lps stimulation	[218]
*Casp-1* ^*−/−*^	C57BL/6J	liver damage in HS model	altered regulation of cell death	[203]
*Casp-1* ^*−/−*^	C57BL/6J	altered metabolism	protection form non-alcoholic steatohepatitis, atherosclerosis, obesity (age and sex-dependent manner)	[198, 219, 220]
*Casp-1* ^*−/−*^	C57BL/6J	abnormal reaction to IAV	induction of more severe pneumonia by iav, increased replication rate	[208]
*Casp-1* ^*−/−*^ *11* ^*−/−*^ *12* ^*−/−*^	C57BL/6	no overt abnormalities	no abnormalities in apoptosis, no abnormalities in septic shock (compared to *Casp-1*^*−/−*^*11*^*−/−*^)	[236]
*Casp-11* ^*−/−*^	C57BL/6J	normal development	fibroblast resistance to apoptosis, resistance to lethal dose of lps	[221]
*Casp-11* ^*−/−*^	C57BL/6J	reduced apoptosis in stroke model	defect in caspase-3 activation	[223]
*Casp-11* ^*−/−*^	C57BL/6J	resistance to allergic lung inflammation	decreased levels of leukocytes in bronchoalveolar lavage fluid, fewer infiltrating alveolar eosinophils	[229]
*Casp-11* ^*−/−*^	C57BL/6J	increased susceptibility to colitis	impaired il-18 production, epithelial barrier, and proliferation	[232]
*Casp-11* ^*−/−*^	C57BL/6	altered response to bacterial infection	decreased macrophage cell death, protection from lethal dose of lps	[218]
*Casp-11* ^*−/−*^	C57BL/6	abnormal host-defence response	decreased cell migration mediated by actin depolymerization	[234]
*Casp-11* ^*−/−*^	C57BL/6	sensitivity to colitis-associated carcinogenesis	decreased stat1 activity	[233]
*Casp-12* ^*−/−*^	129 X C57BL/6J	normal development	resistance to ER stress-induced apoptosis, defective apoptosis of cortical neurons induced by amyloid-beta protein	[239]
*Casp-12* ^*−/−*^	C57BL/6J	resistance to peritonitis and septic shock	dampened production of ifnγ	[243]
*Casp-12* ^*−/−*^	C57BL/6J	less severe colonic inflammation	enhanced production of antimicrobial peptides	[254]
*Casp-12* ^*−/−*^	C57BL/6J	greater mortality in MNV	higher viral burden and defective type i ifn response	[255]
*Casp-12* ^*−/−*^	C57BL/6J	enhanced malaria clearance at blood-stage	enhanced nf-κb activation (via pathway nemo-iκb kinase complex - nf-κb)	[256]
*Casp-12* ^*−/−*^	C57BL/6J	severe liver pathology during malaria infection	enhanced pro-inflammatory response (not sufficient to overcome infection)	[257]
*Casp-12* ^*−/−*^	C57BL/6J	reduced CCl4-induced hepatic apoptosis	attenuation of activation of caspase-9 and -3	[251]
*Mdx-Casp-12* ^*−/−*^	C57BL/6J	preserved muscle function in *mdx* model	*recovery of specific force generation and resistance to muscle fibre degeneration*	[250]
*T17M/Casp-12* ^*−/−*^	C57BL/6J	preserved vision in retinal pathogenesis	postponed photoreceptor cell death and preservation of retinal structural integrity	[249]
*Casp-12* ^*−/−*^	BL6	obesity and insulin resistance	abnormal nlrp3 inflammasome pathway	[258]

#### Caspase-1

Caspase-1 is the best characterized caspase playing an essential role in inflammation [190]. Caspase-1 activation takes place in assembly of multi-protein complex called inflammasome, which is stimulated by several small molecules derived from infection, tissue damage, or metabolic dysfunctions. There are many types of inflammasomes, where NLR families are the most common responsible for host immune responses against infection, trauma or tissue necrosis [191].

Caspase-1 acts on the cleavage of downstream substrates, including the maturation of the inflammatory cytokines, IL-1β and IL-18, which are among its most important functions [192]. In addition, caspase-1 activation occurs in pyroptosis, a rapid caspase-1-dependent form of cell death frequently induced by infected macrophages. During this process, cleavage of gasdermin D occurs, serving as a pore-forming protein in the formation of channels for secretion of IL-1β and IL-18 [192, 193]. Some authors also suggest a role for caspase-1 in apoptosis [192]. Further, caspase-1 is present in a variety of cell types and is involved in numerous cellular processes such as myoblast differentiation and fusion to multinucleated myotubes [194], neural cell differentiation, or chondrogenesis [195, 196]. Caspase-1 was also associated with the regulation of glucose and lipid metabolism [197], making it a potential target molecule in the treatment of metabolism-related disorders, such as obesity [198], diabetes or osteoarthritis [199], cancer, and non-alcoholic fatty liver disease [200]. Caspase-1-deficient mice are born live with no apparent spontaneous developmental defects [201].

#### Apoptotic effect of caspase-1 deficiency

Caspase-1-deficient mice did not show major defects in apoptosis [201, 202] but manifested higher levels of liver damage, cell death, and neutrophil influx in haemorrhagic shock. This phenotype indicated hepatoprotective role of caspase-1, due to its ability to regulate cell death pathways by binding anti-apoptotic proteins Bcl-2 and Bcl-xL [203].

In contrast, caspase-1-deficient mice displayed a significant reduction in mortality after myocardial infarction suggesting a pro-apoptotic role of caspase-1 in the heart. When considering the underlying mechanism, caspase-1 was suspected to cleave caspase-9 and -3, but not caspase-8, indicating activation of the intrinsic apoptotic pathway [204]. This correlates with study where caspase-1-deficient neutrophils were susceptible to Fas-mediated apoptosis. Further, delayed LPS-mediated apoptosis was observed in WT neutrophils but not in those deficient in caspase-1 [205]. A pro-apoptotic effect was observed in studies involving retinal neurons injured by excessive light exposure and I/R, where reduced apoptosis was observed [206], as well as in a model of colitis-associated colorectal cancer [207].

#### Non-apoptotic effect of caspase-1 deficiency

Given the major role assigned to inflammation, caspase-1-deficient mice display distinct reactions when exposed to viral and bacterial stimuli in comparison with WT mice. For instance, upon challenge with IAV, caspase-1-deficient mice exhibited a 40% mortality rate, contrasting with the 10% observed in WT mice, leading to severe diffuse alveolar damage in the lungs of caspase-1-deficient mice [208]. This increased susceptibility to IAV infection was associated with decreased cytokine production [209]. Similarly, the absence of caspase-1 led to increased susceptibility to *Salmonella typhimurium* infection [210]. Conversely, treatment of caspase-1-deficient mice with LPS injection resulted in survival advantage compared to WT mice [202], and an improved clinical status was was observed in caspase-1-deficient mice with *Pneumococcal meningitis* and *Pseudomonas aeruginosa* corneal infection [211, 212]. These findings suggest that caspase-1 operates specifically in response to various stimuli and individual cell characteristics should also be taken into account.

Mice deficient for caspase-1 were defective in the secretion of IL-1β, IL-18, or pro-IL1α [201, 202]. Due to the inability to process pro-IL-18, caspase-1-deficient mice injected with LPS exhibit defective interferon (IFNγ) production. Since IFNγ is an important regulator of cell proliferation, caspase-1-deficient mice show a higher proliferation rate in splenocytes after LPS stimulation [213]. Altered levels of pro-inflammatory cytokines were also observed in other organs and tissues affected by various insults. For instance, in acute renal failure (ARF)/acute tubular necrosis (ATN), caspase-1-deficient mice display an improved phenotype compared to WT mice [214–216]. These mice do not show the increase in IL-18 observed in WT mice during ARF; instead, they exhibit decreased neutrophil infiltration [214]. Furthermore, lower brain IL-1β levels protect caspase-1-deficient mice from ischemia [217, 218].

Besides changing of inflammatory status, caspase-1 deficiency also resulted in increased proliferation of colonic epithelial cells in a model of colitis-associated colorectal cancer [207].

Another category of caspase-regulated processes is the metabolism. Caspase-1-deficient mice develop obesity depending on age and sex when kept on high-fat diet. This phenotype was attributed to lower levels of IL-18, as IL-18-deficient mice show a similar tendency [198]. The absence of caspase-1 further decreased the harmful effect of high fat diet on the liver [219]. Moreover, caspase-1 deficiency improved the phenotype in atherosclerosis-prone apolipoprotein E-deficient (Apoe^−/−^) mice displaying poor lipoprotein clearance, resulting in atherosclerotic plaques. In this case, caspase-1 promoted atherosclerosis by enhancing the inflammatory status of the lesion [220].

#### Caspase-11

The functions of caspase-11 remain unclear. While its expression in healthy mice was low, it is highly inducible upon different stimuli [12], including injection of LPS [221]. Unlike other caspases, caspase-11 requires a transcription-dependent signal to up-regulate its cellular expression prior to its activation [12]. In contrast to caspase-1, caspase-11 activation does not require an upstream sensory complex and can be directly activated by LPS [222]. Despite being classified as an inflammatory caspase, it also shares some characteristic with initiator group [223]. The main function of caspase-11 is the induction of non-canonical pathway of pyroptosis [224]. Once this process is activated, caspase-11 cleaves the major substrate protein gasdermin D [225]. Unlike caspase-1, caspase-11 cleaves gasdermin D independently of inflammasome mediators [12, 223]. Caspase-11 also participates in apoptosis [226] where it cleaves caspase-3 [223]. Additionally, it regulates autophagy in response to bacterial insults [227] and modulates intracellular trafficking by influencing of actin polymerization and cell migration [228]. Furthermore, caspase-11 was revealed to play a role in the pathophysiology of asthma and allergy [229]. It also can be involved in brain injury-induced neuronal pyroptosis [230]. Caspase-11-deficient mice are born live without significant developmental defects [221].

#### Apoptotic effect of caspase-11 deficiency

Caspase-11 exhibited reduced population of apoptotic cells after being subjected to middle cerebral artery occlusion, a mouse model of stroke [223]. The decreased apoptosis was assigned to decreased activation of caspase-3. Caspase-11 further contributed to macrophage death during *Salmonella typhimurium* infection [231]. Importantly, the process was not dependent on IL-1β/IL-18 maturation and caspase-11 was shown activated in non-canonical inflammasome during this process.

#### Non-apoptotic effect of caspase-11 deficiency

Caspase-11-deficient mice were found to be protected from sepsis induced by LPS, and they manifested defective secretion of interleukins [221]. Based on this observation, caspase-11 was indicated to interact with caspase-1 and promote its activation [221]. Since caspase-1 and caspase-11 are located close to each other on the chromosome, caspase-1-deficient mice also lacked caspase-11, making it difficult to separate their functions. Further studies using genetically targeted mice provided insight into the specific roles of caspase-11. Thus caspase-11, rather than caspase-1, may be the critical effector of deleterious inflammatory responses [218].

Caspase-11-deficient mice manifested increased susceptibility to inflammatory disease such as colitis due to impaired IL-18 production, resulting in reduced intestinal epithelial barrier integrity and decreased cell proliferation [232]. Additionally, they were more sensitive to colitis-associated carcinogenesis, showing increased expression of proteins associated with early-stage of angiogenesis. The heightened susceptibility of caspase-11-deficient mice was associated with decreased Signal Transducer and Activator of Transcription 1 (STAT1) activity [233]. On the other hand, caspase-11-deficiency conferred protection from allergic lung inflammation. These mice showed decreased levels of leukocyte numbers in bronchoalveolar lavage fluid and had fewer infiltrating alveolar eosinophils [229].

Caspase-11 was suggested to play role in regulation of lymphocyte migration during inflammation. Caspase-11 interacts with actin interacting protein 1 (Aip1), an activator of cofilin-mediated actin depolymerisation [234].

#### Caspase-12

Despite being initially classified as an inflammatory caspase, caspase-12 function has not yet been sufficiently explained [235, 236]. Caspase-12 differs from inflammatory caspase-1 and -11 in several aspects. It does not participate in the maturation of IL-1β and is not present in macrophages, which are typical models of inflammatory cells [237]. Some authors speculate about its function in cell death induced by ER stress, which frequently occurs due to the accumulation of misfolded proteins and changes in calcium homeostasis [238, 239]. Activation of caspase-12 was detected in some models of apoptotic induction [240, 241], while in others, it was not [239, 242]. Some studies documented a suppressive effect of caspase-12 on caspase-1, which would then enhance vulnerability to sepsis [243]. However, this function of caspase-12 has also been questioned [244]. These contradictory scientific outcomes make caspase-12 difficult to classify and characterize its physiological function. Furthermore, the activation of caspase-12 is not fully understood. In some circumstances, it has been observed to be activated by calpain, TRAF2, and caspase-7 [245–247].

Caspase-12 was detected in various tissues during development, but its constitutive expression was associated with only some cell types, such as epithelia or primary fibroblasts [180, 240, 245]. Interestingly, caspase-12 was found in developing bone and may regulate the expression of osteogenic markers such as *Alpl*, *Bglap*, and *Phex* [248]. Mice, unlike humans, express full length caspase-12 which can undergo proteolytic cleavage [240]. Multiple roles of caspase-12 thus were hypothesized in mice. In humans, most people express truncated form of caspase-12 lacking catalytic domain and only about 20% African descent people express full length protease which is a risk factor for developing sepsis [14]. From the clinical aspect, caspase-12 has shown potential in the treatment of inherited retinopathy [249] and Duchenne muscular dystrophy (DMD) [250]. Additionally, it is speculated to play a role in neurological diseases due to its putative engagement as ER stress sensor [239]. Remarkably, caspase-12-deficient mice are born live without significant developmental defects [239].

#### Apoptotic effect of caspase-12 deficiency

Engagement of caspase-12 in apoptosis was dependent on different stimuli [239]. The reduction of apoptosis as observed in different disease model in caspase-12-deficient mice could be either beneficial or harmful for treatment of pathologies [239, 249, 251]. Caspase-12-deficient cortical neurons were defective in ER apoptosis induced by amyloid-β protein and thus caspase-12 may contribute to amyloid-β neurotoxicity [239]. Mechanism of ER induced apoptotic pathway was not understood. One of the hypotheses speculates that an imbalance in Ca^2+^ homeostasis can cause calpain translocation to the ER leading to activation of caspase-12 [252]. Caspase-12 then induces the caspase-3-dependent apoptotic pathway through the activation of caspase-9 [253].

Caspase-12 ablation in T17M retinas (model of retinal pathology) resulted in postponed photoreceptor cell death and preservation of retinal structural integrity with the scenarios where ER stress-IRE1-TRAF2-Csp12-Csp3/7 and the calcium-induced active calpain-caspase-12-Csp-3/7 pathways contribute to retinal pathogenesis in T17M mice through activation of caspase-12 [249].

In carbon tetrachloride-induced hepatocytes, reduced apoptosis was observed in caspase-12-deficient mice compared to WTs, resulting in decreased liver damage. This phenotype was accompanied by attenuated activation of caspase-9 and -3, supporting caspase-12 action on caspase-3 directly and/or indirectly *via* caspase-9 activation [251].

#### Non-apoptotic effect of caspase-12 deficiency

Similar to its apoptotic functions, the non-apoptotic roles of caspase-12 were dependent on specific cell types and stimuli. Caspase-12-deficient mice have been observed to have increased resistance to polymicrobial sepsis and peritonitis [243], as well as to some bacterial infections [254], when compared to WT mice. The survival advantage of the caspase-12-deficient mice resulted from more efficient clearance of bacterial infection than in WT littermates. This was accompanied by increased levels of pro-inflammatory cytokines, including IFNγ, which was critical for the process [243]. Consistently, improved pathogen clearance was associated to NF-κB activation [254].

In contrast to bacterial infection, caspase-12-deficient mice exhibit greater mortality during West Nile virus (WNV) infection compared to WT mice. This was accompanied by exacerbated neurological symptoms, higher viral burden and defective IFNβ response [255]. Despite increased level of pro-inflammatory cytokines [256], caspase-12-deficient mice were not universally protected from malaria infection [257].

Higher level of obesity was observed on a high-fat diet in caspase-12-deficient mice compared to their WT counterparts. They increased liver weight, serum cholesterol, liver triglycerides and elevated liver damage. They also developed glucose intolerance and insulin resistance. This phenotype might be dependent on the NLRP3 inflammasome, since *Casp12*^*−/−*^*Nlrp3*^*−/−*^ mice did not develop obesity and were similar with WT mice [258].

Deletion of caspase-12 improved phenotype of *mdx* mice, model for DMD. ER stress is heightened in dystrophic muscles and contributes to the pathology of DMD. *Mdx*^*−/−*^*Casp-12*^*−/−*^ mice had a 75% recovery of both specific force generation and resistance to eccentric contractions. The compensatory hypertrophy normally found in *Mdx*^*−/−*^ muscles was normalized when caspase-12 was deleted. The mechanism by which caspase-12 deletion preserves *Mdx*^*−/−*^ muscle function is not known. Possible mechanisms may include an improvement in regeneration, protection of contractile proteins from degradation but also apoptotic aspect cannot be excluded [250].

### Caspase with differentiation function

The phenotypes of mice deficient for caspase-14 are listed in Table 6.

**Table 6:** Phenotypes of mice lacking caspase-14

	mouse strain	development/ phenotype	apoptotic/ non-apoptotic/unspecified effect	reference
*Casp-14* ^*−/−*^	Swiss Webster X 129	shinier and more lichenified skin	abnormal cleavage of profilaggrin	[266]
*Casp-14* ^*−/−*^	Swiss Webster X 129	reduced epidermal barrier	defect in the terminal filaggrin degradation	[260]
*Casp-14* ^*−/−*^	Swiss Webster X 129	predisposed parakeratosis	abnormal differentiation and the maintenance of stratum corneum	[268]
*Casp-14* ^*−/−*^	Swiss Webster X 129	enhanced antibacterial response	imbalance of the skin-resident bacterial communities	[269]

#### Caspase-14

Caspase-14 stands out as a unique member of the caspase family, distinct from both apoptotic and inflammatory groups of caspases. Activation of caspase-14 primarily occurs in epithelial cells of the skin and hair follicles undergoing a special type of cell death called cornification [259]. Its crucial role lies in the processing of profilaggrin to filaggrin and later into hygroscopic amino acids, which act as one of the elements of natural moisturizing factors, thereby contributing to the maintenance of the skin barrier against water loss [260]. The clinical relevance of caspase-14 is particularly evident during the terminal differentiation of skin keratinocytes and the maintenance of normal stratum corneum [261]. In contrast to other caspases expressed ubiquitously in various cells, caspase-14 was located specifically in cornifying epithelia and hair follicles, Hassall’s bodies of the thymus gland, and in the forestomach of rodents [259, 262]. Although the regulation of the caspase-14 gene has not been fully elucidated, it is speculated to be tightly connected with processes of epidermal differentiation [263].

Furthermore, caspase-14 expression has been described in various types of cancer and diabetic retinopathy [263] In the context of the skin diseases, increased expression of caspase-14 has been found in cancerous lesions, while decreased expression was associated with psoriasis or atopic dermatitis [264, 265]. Notably, caspase-14-deficient mice born live, are fertile, and live as long as WT mice [260, 266].

#### Apoptotic effect of caspase-14 deficiency

Caspase-14 was not associated with activation in response to apoptotic stimuli [267].

#### Non-apoptotic effect of caspase-14 deficiency

Caspase-14 deficiency particularly impacted skin cornification [263]. The skin of new-born caspase-14-deficient mice was shinier and more lichenified than in WT mice [266]. Furthermore, caspase-14-deficient mice have decreased epidermal hydration, higher transepidermal water loss, and three times lower levels of natural moisturizing factors [260, 266]. This is because, in caspase-14-deficient mice, processing of profilaggrin, the only known substrate of caspase-14, into fillagrin was initiated but not completed, resulting in the accumulation of filaggrin fragments, which leads to various aberrant phenotypes [260].

The skin of caspase-14-deficient mice was also more sensitive to different stimuli compared to WT mice. Following repetitive treatment by acetone, a higher incidence of large parakeratotic plaques was observed in caspase-14-deficient mice compared to WTs [268]. Additionally, the skin of caspase-14-deficient mice exhibited heightened sensitivity to the formation of cyclobutene pyrimidine dimers after UVB irradiation, resulting in increased levels of UVB-induced apoptosis [266]. Furthermore, caspase-14 ablation resulted in an increase in bacterial richness and diversity during steady-state conditions and caspase-14-deficient mice showed enhanced antibacterial response compared to WT mice when challenged with bacteria [269].

#### Comparison of knockout animals

Mice deficient in caspases displayed some interesting phenotype similarities. The most apparent resemblance was seen in decreased elimination of neurons, resulting in excessive neural tissue incompatible with life in caspase-3 or -9-deficient mice [69, 131, 270]. A similar observation was made in Apaf-1 deficient mice [271], highlighting the crucial role of the intrinsic apoptotic pathway for neural development and viability. Caspase-8-deficient mice also exhibited abnormalities of neural system; however, the lethality observed in caspase-8 null mice resulted from uncontrolled necroptosis [101]. Therefore, the extrinsic pathway likely plays a minor or specific role in this process. Mice lacking the caspase-3 or -9 (or also Apaf-1) suffered from anatomic and functional abnormalities of the inner ear, which was again attributed to the decreased levels of the intrinsic pathway of apoptosis [116, 133, 144].

Insufficient apoptosis further impacted caspase-2 or -9-deficient ovaries [46, 125], albeit with different timing. Conversely, deficiency of the anti-apoptotic Bcl-2 resulted in decreased oocyte population [272], further supporting the importance of mitochondria in this process. Surprisingly, all caspase-deficient mice, except caspase-14, showed some alterations in apoptosis depending on the stimuli or cell types (see Tables 3, 4 and 5). This suggests existence of various pathways that may be employed in specific cells and situations.

Deficiency of inflammatory caspases resulted in altered response to various types of infection, with either beneficial or deteriorative effects for the mutant mice (see Table 5). As observed in caspase-1 or -11-deficient mice, the phenotype may result from impaired cytokine production [202, 232]. On the contrary, caspase-12-deficient mice showed an increased pro-inflammatory response [257], highlighting the specific character of caspase-12.

In terms of non-lethal functions, caspase-3 or -7 deficiency has been linked to abnormalities in differentiation and the formation of hard tissues [30, 31, 179]. Although both deficient models have shown alterations in osteogenic gene expression [30, 179], the function of these caspases may also involve the degradation of stem-cell-specific factors [43, 188]. Caspase-3 or -12 deficiency have been implicated in skeletal muscle function [29, 250], but unrelated mechanisms were suggested to be involved. Caspase-1, -3 or -8 have been observed to regulate the proliferation [102, 134, 150, 207] of various cell types, making it difficult to determine a general mechanism for this process. Additionally, caspase-1, -2 or caspase-12 deficient mice have shown alterations in metabolism and the development of obesity, with specificities for each caspase [76, 198, 258]. Impaired synaptic formation and plasticity have been observed in caspase-9-deficient [122] or caspase-3-deficient mice [154, 155]. Furthermore, behavioural alterations have been detected in caspase-3 or caspase-6-deficient mice [154, 155, 163]. While caspase-1 or -2-deficiency has been associated with increased susceptibility to tumor induction [75, 207], deficiency of caspase-3 has been linked inversely to a decreased incidence of cancer [147]. These data highlight the diverse roles of caspases, not only as tumour inhibitors but also as tumour inducers, across various pathways. In general, the apoptotic pathway seems to be more conserved compared to non-apoptotic mechanisms, and disruption of the apoptotic function of caspases results in more devastating effect compared to abnormalities resulting from their non-apoptotic roles.

#### Future perspective of caspase research

Caspases have been recognized as great promising targets for treating various human diseases [273–275]. However, the efficacy, specificity, and side effects of pharmacological caspase inhibitors, the primary tools in clinical studies and future applications, remain significant challenges [275]. Despite recent advancement such as nanoparticle delivery systems and CRISPR/Cas9 gene editing showing promising potential in caspase treatment [276, 277], many questions about caspases themselves remain unanswered. The main challenge lies in describing and understanding the multiple and sometimes contradictory functions of caspases. This is closely related to further identification of their substrates and downstream pathways, including caspase regulators. Notably, the switch between lethal and non-lethal functions is considered a fundamental question, with several mechanisms proposed to regulate the process, such as subcellular localisation [278], availability of specific substrates [279], compensation by anti-apoptotic proteins [280, 281], various levels of activation [282], the biological status of cells (cell type, state of growth/differentiation), cellular context (environment), caspase activation vs. non-activated state, and mechanisms of activation. Many of these aspects may be further explored using mouse models, with temporal (Tamoxifen-inducible gene deletion) and/or spatial regulation (Cre/loxP recombination system) of caspase deficiency helping to specify caspase engagement in the development of different organs. However, it is important to note that while mouse models are valuable, there are limitations to directly extrapolating their findings to human research. For instance, caspase-8 deficiency is incompatible with life in mice but only results in immunological disorders in human [283]. Finally, the focus of the field should be on translational work and complementing mechanistic models drawn from experimentation in mice.

## Conclusion

Mouse caspase models has greatly contributed to the progress in explaining the traditional but also emerging roles of these cysteine-aspartate proteases in the last decades. We hope that this comprehensive overview of available mouse models will not only acknowledge their hitherto utility but also emphasize their importance in enhancement of the development within this research field associated with basic as well as applied research.

## Data Availability

No datasets were generated or analysed during the current study.

## Abbreviations

AD: Alzheimer disease
ADHD: Attention deficit/hyperactivity disorder
Aip1: Actin interacting protein 1
Apoe: Apolipoprotein E-deficient
APP: β-amyloid precursor protein
ARF: Acute renal failure
ATN: Acute tubular necrosis
Bcl-2: B-cell lymphoma 2
CAD: Caspase-activated DNase
CARD: Caspase-activation recruitment domain
Casp: Caspase
CED: Cell death protein
d.p.c.: Day post coitum
DED: Death effector domain
DISC: Death-inducing signalling complex
DMD: Duchenne muscular dystrophy
DR: Death receptor
DRG: Dorsal root ganglion
ER: Endoplasmic reticulum
HD: Huntington disease
HFSC: Hair follicle stem cells
HS: Haemorrhagic shock
I/R: Ischemia/reperfusion
IAP: Inhibitor of apoptosis protein
IAV: Influenza A virus
ICE: Interleukin-1β-converting enzyme
IFN: Interferon
IL: Interleukin
JNK: C-Jun N-terminal kinase
LPS: Lipopolysaccharides
MEF: Mesenchymal embryonic fibroblasts
MLKL: Mixed Lineage Kinase domain-Like
MST1: Mammalian Sterile Twenty-like kinase
NF-κB: Nuclear factor κB
NGF: Nerve growth factor
NLRP3: NOD-, LRR- and pyrin domain-containing protein 3
ON: Optic nerve
PEK: Primary enamel knot
RGCs: Retinal ganglion cells
RIP1: Receptor-interacting protein 1
RIPK3: Receptor Interacting Serine/Threonine Kinase 3
STAT1: Signal Transducer and Activator of Transcription 1
WNV: West Nile virus
WT: Wild type
YAP: Yes-associated protein
ZBP1: Z-DNA-binding protein 1
